# An artificial habitat increases the reproductive fitness of a range-shifting species within a newly colonized ecosystem

**DOI:** 10.1038/s41598-019-56228-x

**Published:** 2020-01-17

**Authors:** Zachary J. Cannizzo, Susan Q. Lang, Bryan Benitez-Nelson, Blaine D. Griffen

**Affiliations:** 1 Marine Science Program, School of the Earth, Ocean, and Environment, University of South Carolina, Columbia, SC 29208 USA; 2 Geology Program, School of the Earth, Ocean, and Environment, University of South Carolina, Columbia, SC 29208 USA; 3 Department of Biology, Brigham Young University, Provo, UT 84602 USA; 4 Present Address: National Oceanic and Atmospheric Administration Office of National Marine Sanctuaries – National Marine Protected Areas Center, Silver Spring, MD 20910 USA

**Keywords:** Climate-change ecology, Community ecology, Population dynamics

## Abstract

When a range-shifting species colonizes an ecosystem it has not previously inhabited, it may experience suboptimal conditions that challenge its continued persistence and expansion. Some impacts may be partially mitigated by artificial habitat analogues: artificial habitats that more closely resemble a species’ historic ecosystem than the surrounding habitat. If conditions provided by such habitats increase reproductive success, they could be vital to the expansion and persistence of range-shifting species. We investigated the reproduction of the mangrove tree crab *Aratus pisonii* in its historic mangrove habitat, the suboptimal colonized salt marsh ecosystem, and on docks within the marsh, an artificial mangrove analogue. Crabs were assessed for offspring production and quality, as well as measures of maternal investment and egg quality. *Aratus pisonii* found on docks produced more eggs, more eggs per unit energy investment, and higher quality larvae than conspecifics in the surrounding salt marsh. Yet, crabs in the mangrove produced the highest quality larvae. Egg lipids suggest these different reproductive outcomes result from disparities in the quality of diet-driven maternal investments, particularly key fatty acids. This study suggests habitat analogues may increase the reproductive fitness of range-shifting species allowing more rapid expansion into, and better persistence in, colonized ecosystems.

## Introduction

Species range shifts are one of the most widespread symptoms of climate change, occurring across marine^1^, freshwater^2^, and terrestrial habitats^3^. Range shifts alter not only the distribution of species, but also the composition of ecological communities and the functioning and resilience of ecosystems in the face of continued change^1,4^. At times, differential shifting responses lead to community reorganization, including the decoupling of species ranges from those of the foundation species of their historic ecosystems^5^. When this occurs, a shifting species may colonize an ecosystem for which it has no ecological or evolutionary experience (i.e. novel to the colonizing species)^5^ and where novel interactions are likely to result in suboptimal conditions^6–8^. While species may survive in such colonized suboptimal ecosystems, their continued spread and persistence may be hindered. The prevalence of such colonizations is expected to increase^5,9^. Thus, understanding how habitat effects impact the fitness of species in newly colonized ecosystems is necessary for understanding and predicting geographic range-shifts.

Reproductive fitness is central to individual and population success. The importance of reproduction is further magnified during range shifts, as propagule pressure is a primary determinant of success during colonization and expansion^10,11^. Habitat effects alter reproductive potential through a range of environmental and biological factors^12,13^ potentially altering an individual’s contribution to the persistence and expansion of a colonizing population. For shifting species, pockets of favorable habitat that provide conditions which increase reproductive success within suboptimal novel ecosystems could play a key role in the fitness, persistence, and continued expansion of the population. Habitats that replicate the conditions of the historic habitat are particularly likely to provide an increase in reproductive fitness. These “analogous habitats”, so named because they act as ecological analogues to a historically-preferred habitat, are often artificial and provide improved conditions for organisms over a surrounding suboptimal environment^8,14^ [and references therein]. These improved conditions could increase the quantity and quality of reproductive investment compared to the surrounding suboptimal habitat, leading analogous habitats to play an important role in the success of range-shifting species. Given the ubiquity of anthropogenic structures (buildings, power lines, boat docks, etc.) in the modern environment, there are many opportunities for these structures to act as habitat analogues.

The range expansion of the mangrove tree crab *Aratus pisonii* provides an opportunity to examine the reproductive impacts of a suboptimal colonized ecosystem (the saltmarsh), and an analogous habitat embedded within (boat docks), at the leading edge of this range expansion. This semi-terrestrial crab has historically been associated with mangrove forests throughout the neotropics^15,16^. Fresh mangrove leaves are the primary diet of these crabs with animal material taken opportunistically^17^ and preferred when available^18^. Individuals show strong site fidelity to individual trees, rarely traveling more than 25 m from their home tree^19^. *Aratus pisonii* generally reside on mangrove branches where they actively avoid water, except to wet their gills or release larvae, due to high aquatic predation^20^. Crabs even leave ground shelter to climb structure on the rising tide^16,21^ (pers. obs.). However, the northern range expansion of this arboreal crab has recently outpaced that of mangroves resulting in its colonization of salt marshes along the South Atlantic coast of the United States, an ecosystem it had not previously inhabited^22^.

The salt marsh presents *A. pisonii* with a mangrove-free environment and has numerous consequences for the crabs found there. Compared to conspecifics in the mangrove, crabs in the marsh experience inferior foraging and dietary conditions^8^, increased predation^20^, and more variable thermal conditions that are warmer in summer and colder in winter^8,23^. The marsh also alters important aspects of *A. pisonii* behavior by inducing riskier foraging and thermoregulation^8^ as well as altered social interactions^24^ and the loss of site fidelity^19^. Further, crabs in the marsh exhibit smaller average and maximum body size^7,8^ and reduced larval quality^7^ compared to crabs in the mangrove. The sum of these effects suggests the salt marsh is a suboptimal habitat for *A. pisonii*. Yet, *A. pisonii* are also found on boat docks within the salt marsh. Compared to the surrounding habitat, the sturdy vertical structure and canopy-like coverings of docks superficially resemble mangroves. Docks provide a rare shaded habitat within the marsh that results in cooler summer and warmer winter conditions than the surrounding habitat^8,23^. The more favorable thermal conditions provided by docks, and a higher quality diet high in animal material are likely responsible for the larger body size of the crabs found there^8^. Docks also provide improved disturbance refuge^25^ and allow *A. pisonii* to persist further north than elsewhere in the salt marsh ecosystem^23^. By providing conditions superior to the surrounding salt marsh and more similar to the historic mangrove^8^, docks provide both a mangrove analogue and a refuge habitat to *A. pisonii* within the suboptimal novel salt marsh ecosystem. However, the impact of the dock habitat on *A. pisonii* reproduction has not yet been explored. While previous studies have compared the reproductive potential between *A. pisonii* in the mangrove and salt marsh^22^, none have examined the mechanisms behind observed differences, which are critical in order to fully understand the system and inform management. Given the numerous benefits docks provide, they may also increase the reproductive potential of *A. pisonii* over conspecifics elsewhere in the marsh, thereby playing an important role in the success and expansion of this range-shifting species.

We sought to determine if docks increase the reproductive potential of *A. pisonii* within the colonized salt marsh by comparing the quantity and quality of offspring produced in these two habitats and in the historic mangrove. We further explored the mechanisms behind any observed differences in reproductive potential by comparing five metrics between each of the three habitats: (1) the proportion of total body mass invested in reproduction; (2) egg energy content, which impacts larval quality^26^; (3) egg glycogen content, an important constituent of arthropod reproductive investment^27,28^; (4) egg lipids, the most important component of embryonic development^29^; and (5) the fatty acids (FA) that made up the egg lipids, which are critical to offspring quality and provide measures of the quality of both maternal reproductive investment and diet^30,31^. Given the breadth of factors investigated, this study represents a thorough exploration of habitat-specific impacts on the reproductive fitness of a range-shifting species and may thus serve as a model for future studies of shifting populations. Ultimately, we hypothesized that *A. pisonii* found on docks would display quantitatively and qualitatively superior reproduction compared to conspecifics in the surrounding salt marsh as a result of dietary differences between habitats, and that reproductive success on docks would be more similar to that found in the mangrove habitat.

## Results

### Reproductive season

The reproductive season of *A. pisonii* outside of the tropics has never been recorded. While *A. pisonii* reproduction is often described as continuous^16,32^, such studies were performed in the tropics where conditions encourage year-round reproduction^33^. In contrast, we found scarcely any ovigerous females in May and November, and none from December-April. This reproductive season was consistent across habitat, latitude (27.43°N to 29.94°N; Table 1), and over four consecutive years (2015–2018).

**Table 1:** Locations of collection sites. X’s denote which sites were used in each aspect of the study.

Habitat	Site	Lat-Long	Energetic Investment	Egg Quality	Larval Quality
Mangrove	Pepper Park	27°29′42′N 80°18′12″W	X	X	X
Mangrove	Round Island Park	27°33′33″N 80°19′53″W		X	X
Mangrove	Oslo Road	27°35′14″N 80°21′55″W	X	X	X
Mangrove	North Causeway	27°28′28″N 80°19′12″W	X	X	
Mangrove	Bear Point	27°25′48”N 80°17′10″W	X		
Salt Marsh	GTM NERR	30°0′49″N 81°20′42″W	X	X	X
Salt Marsh	Anastasia State Park	29°52′40″N 81°16′32″W	X	X	X
Salt Marsh	Vilano Marsh	29°55′16′N 81°17′57″W		X	X
Dock	Palm Valley	30°07′57″N 81°23′08″W	X	X	X
Dock	Yacht Club	29°53′09″N 81°17′08″W	X	X	X
Dock	Boating Club	29°56′34”N 81°18′31″W		X	
Dock	Vilano Dock	29°56′33″N 81°18′32”W			X

### Demographics

The smallest and average sizes of ovigerous females differed between habitats with salt marsh crabs becoming reproductively active at a smaller size than conspecifics in the dock and mangrove habitats while those from the dock were smaller than those from the mangrove (Saltmarsh: Smallest = 8.0, Avg. = 12.2 ± 1.6; Docks: Smallest = 11.1, Avg. = 17.0 ± 2.2; Mangrove: Smallest = 13.4, Avg. = 18.1 ± 2.5; all comparisons: p < 0.001; Fig. S1; Due to the number of statistical tests performed, here we present only the resulting p-values. See Appendix S1: Table S1 for full statistical output). The size distributions of ovigerous females also differed between habitats (p < 0.002; Fig. S1). There was abundant overlap between the distributions of collected females (Fig. S1).

### Energetic investment

**Figure 1:**
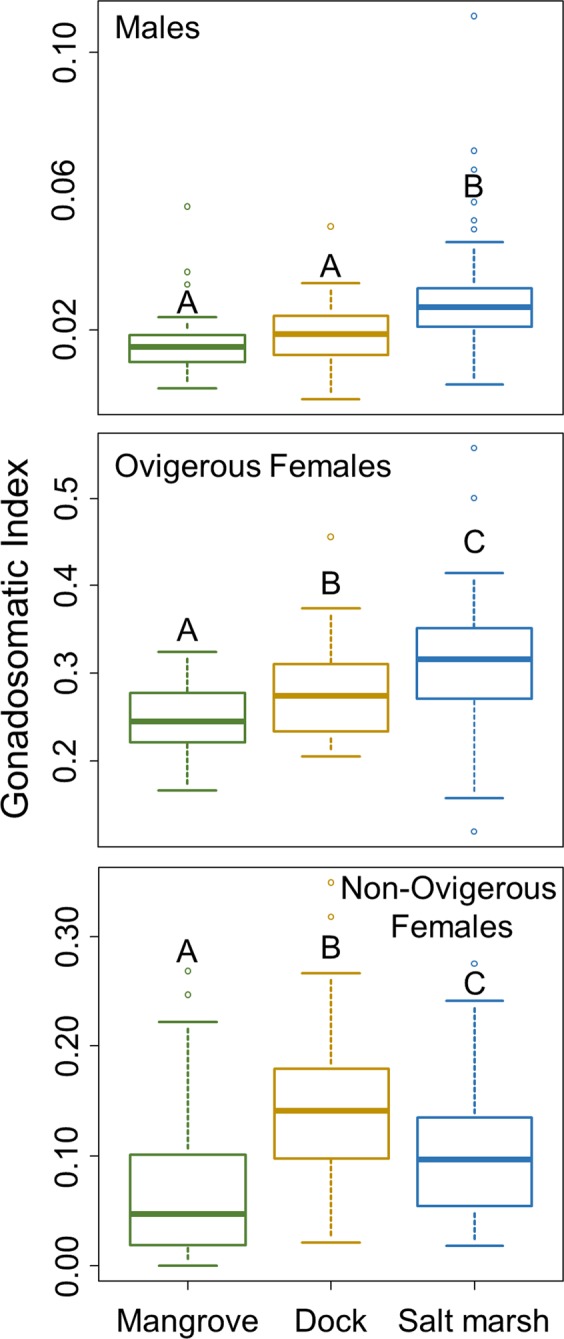
Proportional energetic investment into reproduction, calculated as gonadosomatic index, of male, ovigerous female, and non-ovigerous female *A. pisonii* in different habitats. Letters denote homogeneous groups in this and all other figures presented in this paper. In each boxplot, and in all other boxplots presented in this paper, the median is represented by a heavy line, the box represents the upper and lower quartiles, while the whiskers represent 95% of the data and circles show outliers.

We used the gonadosomatic index (GSI), the proportion of the total dry weight made up of reproductive tissue (eggs and gonads), as a size-independent measure of reproductive effort^34^. Further, as GSI is dependent on sex and reproductive stage, crabs were grouped as males, ovigerous (egg carrying) females, and non-ovigerous females for analysis. While the proportional energetic investment into reproduction does not itself indicate a higher quality reproductive investment or reflect the ultimate quality of a habitat as it relates to reproduction, it is a necessary measurement to understand the ultimate reproductive efficiency of an organism (i.e. the return on investment). The proportion of energy that crabs in these categories invested into reproduction differed between habitats. Crabs from the salt marsh invested a greater proportion of their energy into reproduction than conspecifics of the same sex/reproductive stage from the mangrove, (males: p < 0001, ovigerous females: p < 0.001, non-ovigerous females: p = 0.0162; Fig. 1). Further, in the salt marsh, males and ovigerous females invested proportionally more energy into reproduction than conspecifics on docks (males: p < 0.001, ovigerous females: p = 0.007; Fig. 1) while non-ovigerous females invested proportionally less energy (p = 0.021). Females on docks also invested a greater proportion of their energy into reproduction than conspecifics of the same reproductive stage in the mangrove (ovigerous: p = 0.048, non-ovigerous: p < 0.001) while males from the dock and mangrove did not differ (p = 0.230; Fig. 1).

### Larval quality

**Figure 2:**
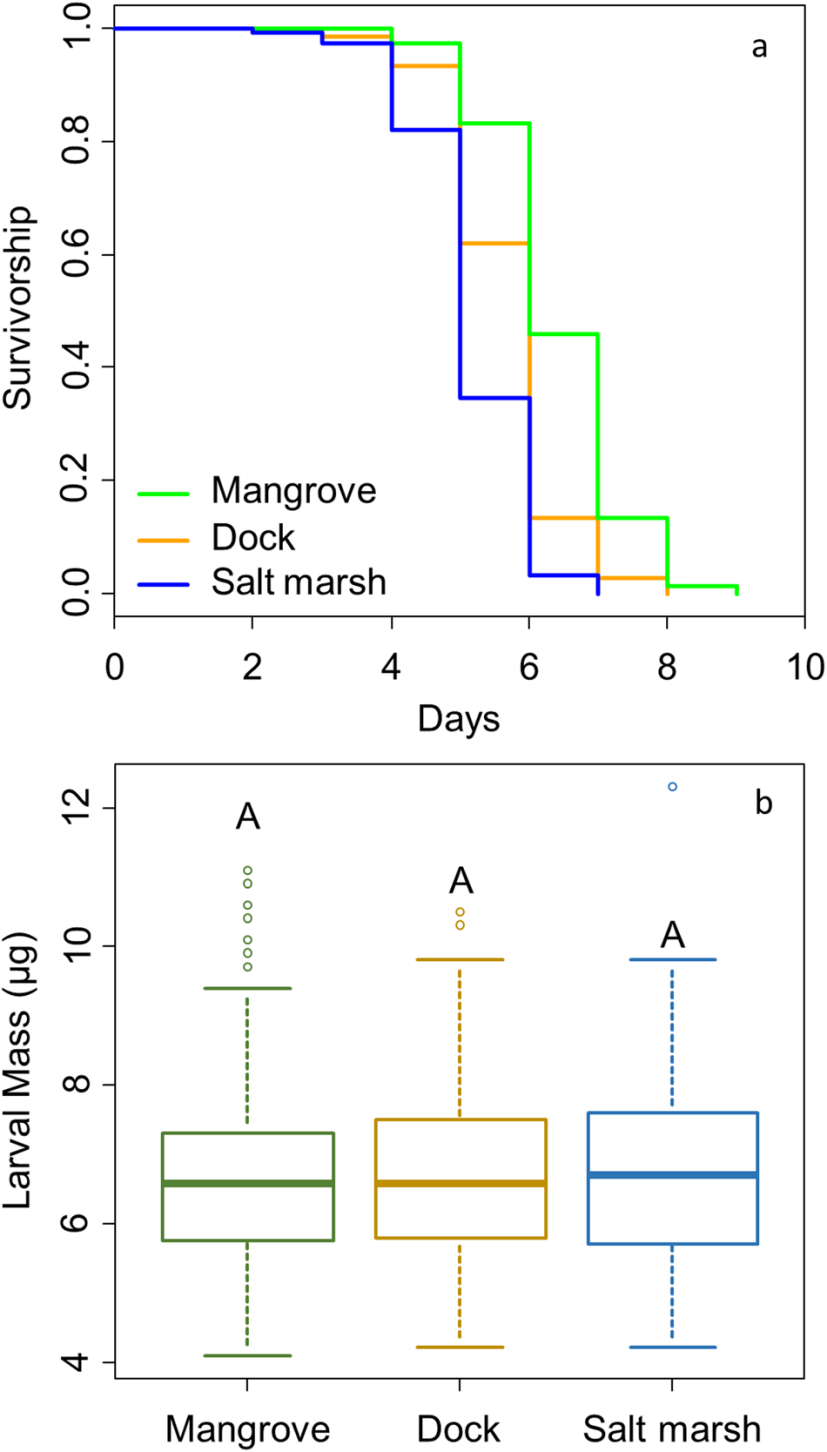
(**a**) Kaplan-Meier curves comparing starvation resistance of *A. pisonii* larvae from different habitats. (**b**) Comparison of larval size at hatching, measured as larval dry mass, between habitats.

We examined larval starvation resistance and larval size (dry weight) upon hatching - both common measures of offspring quality in crustaceans^35–37^. Larval starvation resistance was not impacted by maternal size (p = 0.120) or gut-width:carapace-width ratio (GW:CW; a proxy of long-term diet quality in crabs^38^) (p = 0.880). Thus, these variables were removed to simplify the analysis. Larvae from the mangrove displayed greater starvation resistance than those from either the dock or salt marsh (p < 0.001) while larvae originating from docks showed greater starvation resistance than those from the salt marsh (p = 0.009, Fig. 2a).

Larval size at hatching did not differ between habitats (dock vs. salt marsh: p = 0.781, dock vs. mangrove: p = 0.604, mangrove vs. salt marsh: p = 0.525; Fig. 2b) and was not affected by maternal size (p = 0.222). However, larval size increased with GW:CW (p = 0.025).

### Clutch size

**Figure 3:**
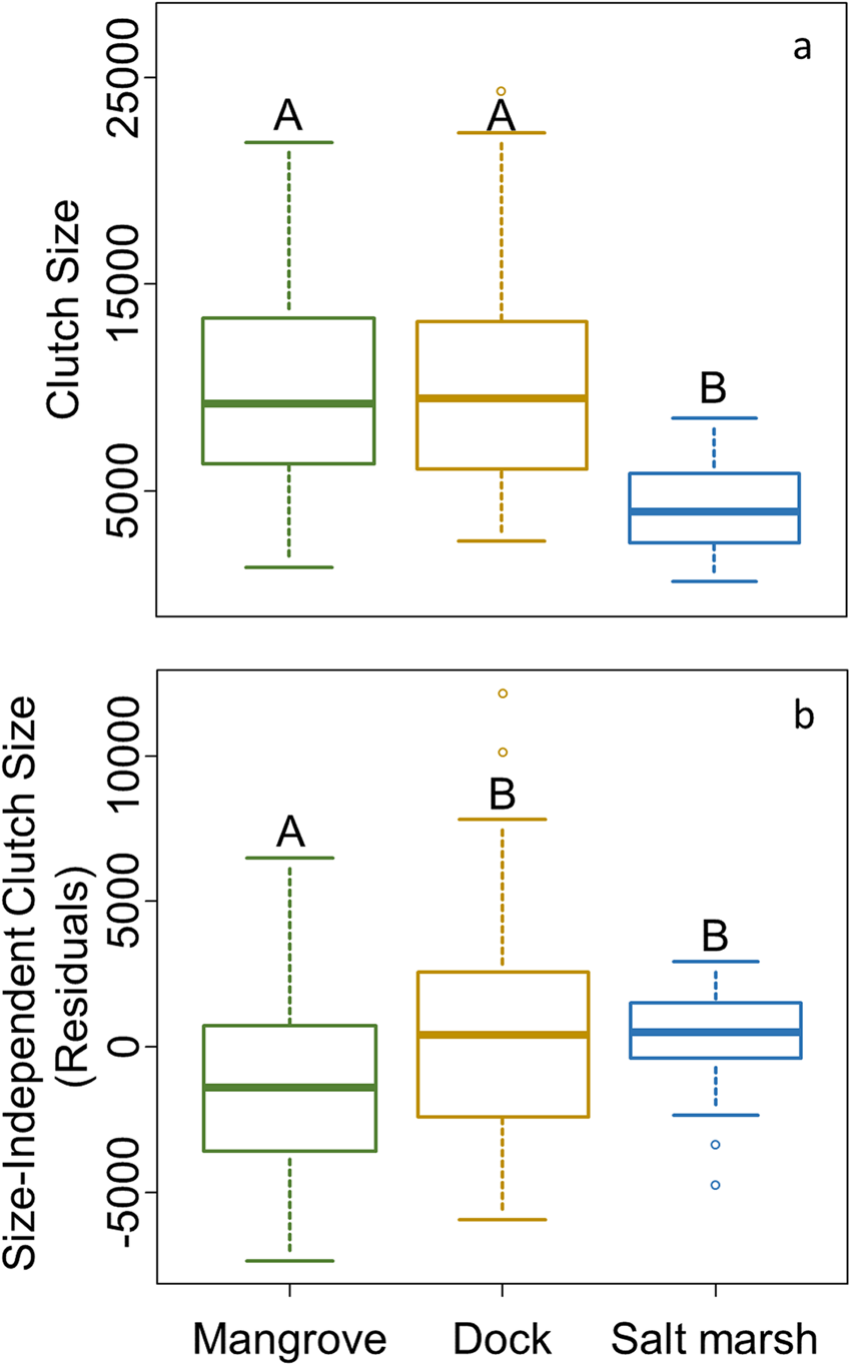
(**a**) *Aratus pisonii* clutch-size in different habitats. (**b**) Size-independent clutch size in different habitats represented by residuals of the relationship between clutch size and crab size (carapace-width).

Crabs from the salt marsh had smaller clutches than conspecifics from either the mangrove or dock habitats (p < 0.001; Fig. 3a), which did not differ (p = 0.994). When clutch size was explored independent of maternal size, it was not impacted by GW:CW (p = 0.275) but crabs produced smaller clutches in October, when clutches were generally smallest, compared to all other months and larger clutches in July, when clutches were generally largest, compared to June and August (p < 0.05). Further, crabs from the mangrove had smaller clutches relative to their body sizes (i.e. size-independent, number of eggs produced per unit size) than conspecifics in the dock and salt marsh habitats (p = 0.038 and 0.034 respectively; Fig. 3b). The size-independent clutch sizes of crabs in the dock and salt marsh habitats did not differ (p = 0.989) despite higher proportional energetic investment (GSI) in the salt marsh.

### Egg energy and glycogen content

Egg energy content was not associated with habitat, month of collection, or maternal variables (p > 0.05, Appendix S1: Table S1, Fig. S2). However, non-eyed (stage-1) eggs had a higher energy content than eyed (stages 2 and 3) eggs (p < 0.001).

Egg glycogen content did not differ between habitats and was not affected by GW:CW or month of collection (p > 0.05, Appendix S1: Table S1, Fig. S3). However, egg glycogen content decreased with increasing maternal size (p = 0.009).

### Egg lipid and fatty acid content

**Figure 4:**
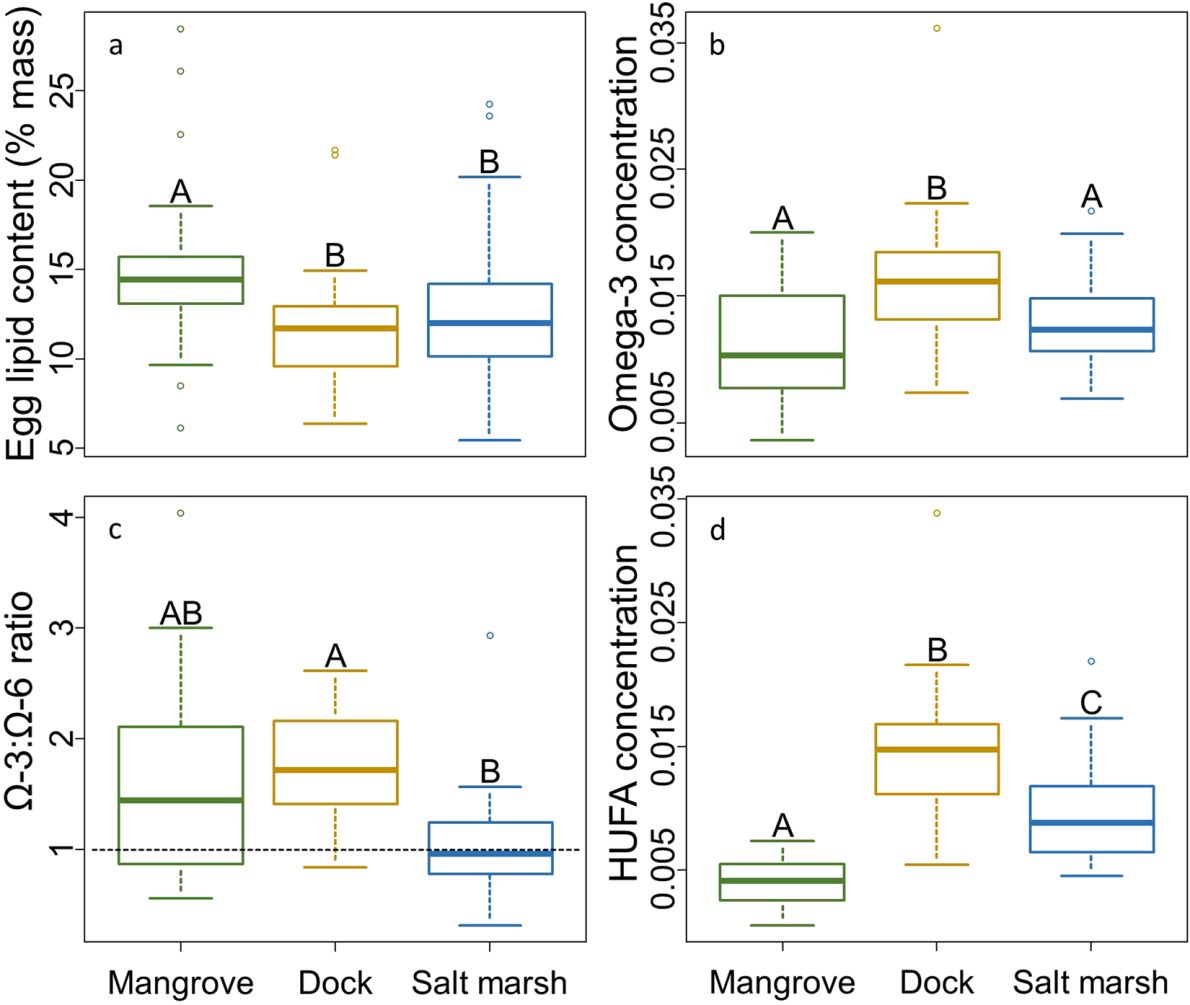
(**a**) Gross lipid content (positively associated with larval quality) of *A. pisonii* eggs originating from each habitat as percent of egg mass. (**b**) Ω-3 fatty acid content (positively associated with larval quality) of eggs originating from each habitat as proportion of egg mass. (**c**) Ω-3:Ω-6 ratio (positively associated with larval quality) of eggs originating from each habitat. Horizontal line represents a 1:1 ratio (**d**) Concentration of HUFA (positively association with larval quality) in eggs originating from each habitat.

*Aratus pisonii* in the mangrove produced eggs with a higher gross lipid content than conspecifics in either the salt marsh or dock habitats (p < 0.001; Fig. 4a), which did not differ (p = 0.725). While egg lipid content was not impacted by maternal variables (size: p = 0.452; GW:CW: p = 0.834), eggs produced in October displayed higher lipid contents than those produced in June and August (p < 0.001). Yet, the lack of an interaction between habitat and collection month (p > 0.05) suggests this seasonal effect was not habitat specific.

Here we present only the results of those FAs and FA groups of particular importance to reproductive potential and larval quality (See Appendix S1: Table S2 for full results). Unless otherwise stated, maternal size, GW:CW, and month of collection had no effect on any FA parameter (p > 0.05). Eggs deriving from the dock habitat had the highest concentration of developmentally important omega-3 FAs (Ω-3s) (vs. mangrove: p < 0.001; vs. salt marsh: p = 0.010; Fig. 4b) including the individual Ω-3s eicosapentaenoic acid (EPA) (vs. mangrove: p = 0.005; vs. salt marsh: p = 0.005) and docosahexaenoic acid (DHA) (vs. mangrove: p < 0.001; vs. salt marsh: p < 0.001). While eggs originating from the mangrove had the lowest concentration of EPA (vs. salt marsh: p < 0.001; vs. dock: p = 0.005), they did not differ from salt marsh eggs in the concentration of overall Ω-3s (p = 0.461) or DHA (p = 0.190). Further, the concentration of the Ω-3 alpha-linolenic acid (ALA) was highest in eggs originating from the mangrove (vs. dock: p < 0.001; vs. salt marsh: p = 0.026) while those from the dock and salt marsh did not differ (p = 0.189). Eggs originating from the dock had the highest concentration of developmentally critical highly unsaturated fatty acids (HUFA, $\,\ge$4 double bonds) with those from the mangrove exhibiting the lowest (dock vs. mangrove: p < 0.001; dock vs. salt marsh: p = 0.002; mangrove vs. salt marsh: p = 0.003; Fig. 4d). Despite their relatively low concentration of Ω-3s, eggs originating from the mangrove had a similar neurogenesis-stimulating omega-3:omega-6 ratio (Ω-3:Ω-6) to those from the dock (p = 0.075; Fig. 4c) and salt marsh (p = 0.564). Eggs originating from docks displayed a higher Ω-3:Ω-6 ratio than those from the surrounding salt marsh (p = 0.046; Fig. 4c). The Ω-3:Ω-6 ratio also increased with increasing maternal size (p = 0.040) resulting in an overall higher ratio in eggs from the mangrove compared to the salt marsh despite the insignificant effect of habitat. There were few seasonal effects, all of which were independent of habitat (habitat*month: p > 0.05), with eggs gathered in October displaying higher HUFA concentrations than those collected in June (p = 0.049) or July (p = 0.009), and higher EPA (p = 0.005) concentrations than those collected in July.

**Figure 5:**
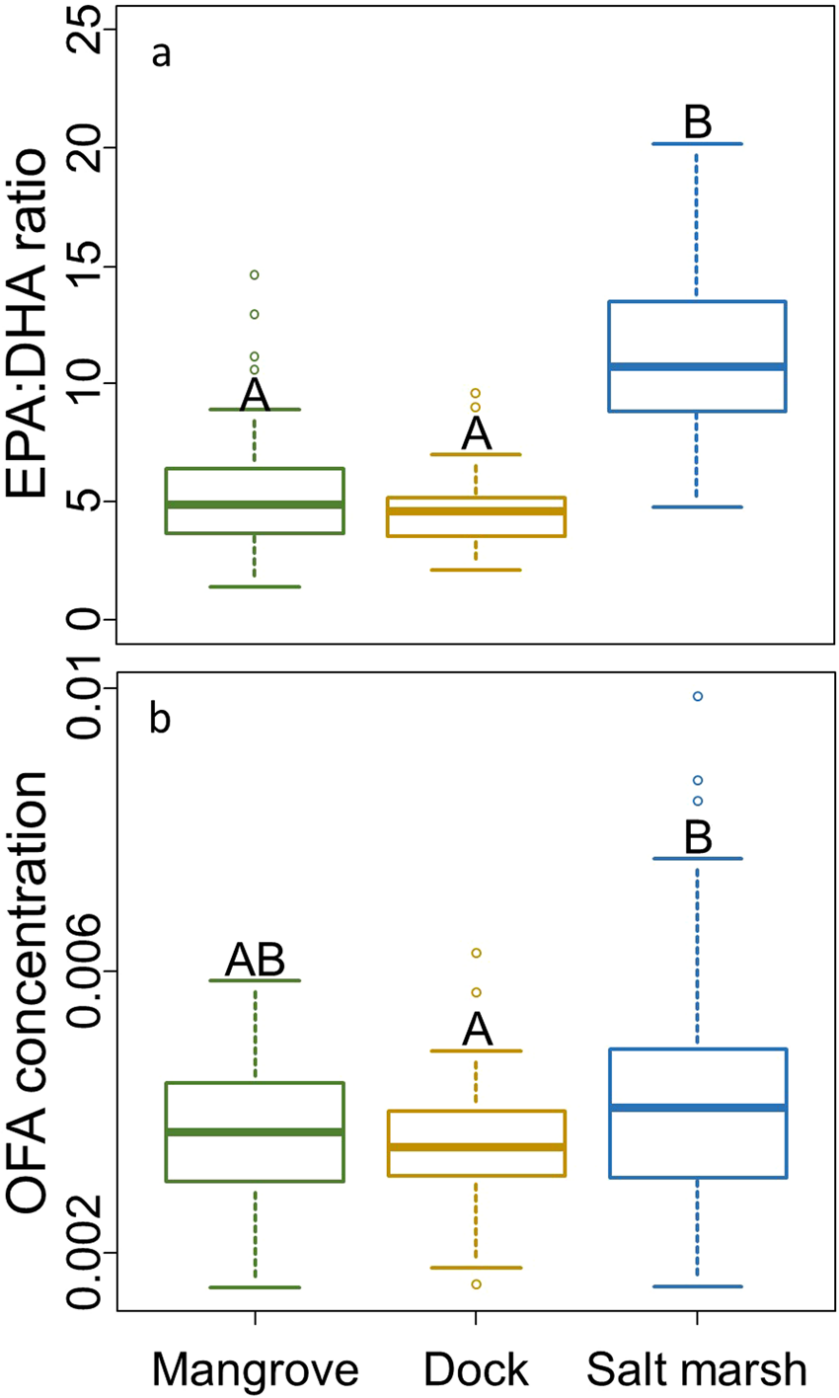
(**a**) EPA:DHA ratio (corresponds positively with maternal trophic level) of eggs originating from each habitat. (**b**) Concentration of odd-numbered fatty acids (corresponds positively with maternal relative detritivory) of eggs originating from each habitat.

Due to the impact of maternal diet on larval quality^12^, we also explored fatty acid trophic markers (FATM). Eggs originating from the salt marsh exhibited the highest EPA:DHA ratio suggesting a lower maternal trophic position^31,39^ than conspecifics in the dock and mangrove habitats (p < 0.001; Fig. 5a), whose eggs did not differ in this measure (p = 0.078). Instead, eggs from the salt marsh displayed higher concentrations of odd-numbered fatty acids (OFAs) than those from docks (p = 0.019; Fig. 5b), suggesting a greater importance of detritus in the maternal diet^31^. The OFA concentration of eggs from the mangrove did not differ from those from the dock (p = 0.157; Fig. 5b) or salt marsh (p = 0.318). Egg OFA concentration also decreased with increasing GW:CW (p = 0.022).

## Discussion

**Figure 6:**
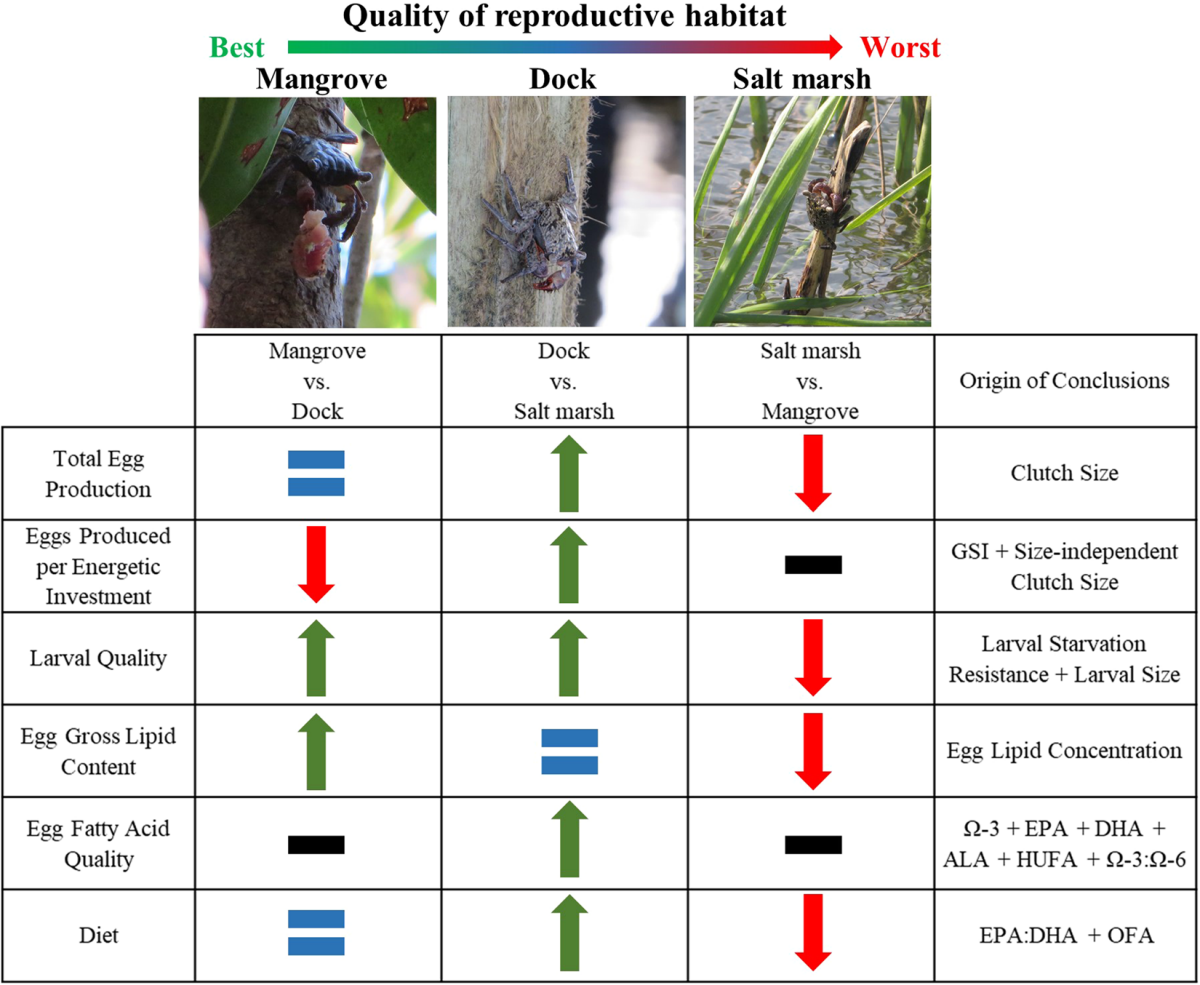
Summary of conclusions drawn from the results of this study. Green arrow indicates the first habitat in the comparison is better for the result being compared while a red arrow indicates it is worse and a blue equal sign indicates the habitats did not differ. A black dash indicates an inability to draw a conclusion.

Our results demonstrate that an artificial analogous habitat within a colonized suboptimal ecosystem can increase the reproductive potential and fitness of a colonizing range-shifting species. Here, this manifests as docks providing a superior reproductive habitat to the surrounding salt marsh. Crabs found on docks produced greater numbers of higher quality larvae for a lower per-egg energetic investment than conspecifics elsewhere in the salt marsh (Fig. 6). Further, the disparity in larval quality appears to be driven by differences in the quality of maternal investment reflected in the egg fatty acids (Fig. 6). While some crabs collected on docks could have spent time in the marsh, this would reduce observed differences making our results conservative and strengthening their explanatory power.

Despite the benefits of analogous habitats, they may remain a subpar reproductive habitat compared to the historic ecosystem of a range-shifter (Fig. 6). In fact, *A. pisonii* in the mangrove produced the highest quality larvae. This is unsurprising, as organisms would be expected to reproduce most successfully under conditions to which they are adapted. However, the higher size-corrected clutch-sizes (i.e. per-size offspring production) of conspecifics in the dock and salt marsh habitats may counteract some of the reproductive fitness lost to larval quality. It is common for individuals in range-edge populations to produce more offspring than conspecifics in the range-core, who tend to apply a strategy of quality over quantity^40^ (and references therein). The higher lipid content of eggs from the mangrove further reflects these differing strategies. Yet, the dock habitat appears to allow *A. pisonii* to straddle these strategies, by producing large numbers of intermediate-quality larvae, and thus reflects a theoretical “mid-range” reproductive habitat despite occurring at the range-edge. Thus, while the historic ecosystem provides the ideal reproductive habitat, the artificial analogue is superior to the surrounding colonized ecosystem. If this pattern holds true across systems, it would provide a general mechanism that facilitates range expansion.

The increased reproductive potential of crabs on docks relative to the surrounding salt marsh emphasizes the potential importance of analogous habitats, and habitat effects in general, to range-shifting species. Egg quality parameters suggest the mechanism behind the acquired benefits as the only measure that differed between the dock and salt marsh was the FA profiles. The FAs invested in eggs are crucial to larval quality^30,41^ and reflect maternal diet^31,42^. Thus, it is likely that more favorable dietary conditions found on docks^8^ are largely responsible for the improved larval quality. This is reflected by eggs from docks exhibiting higher concentrations of the developmentally critical Ω-3s, EPA, DHA, Ω-3:Ω-6^31,41^, and HUFAs^43,44^, all of which indicate a higher quality investment deriving from a high-quality diet^30,42^. The low EPA:DHA ratio of eggs from docks further supports this hypothesis by indicating a higher trophic position^31,39^. This suggests the dietary origin of the improved investment is animal material, a high-quality food source preferred by *A. pisonii*^18^ which is likely an important dietary component on docks^8^. In contrast, the high concentration of OFAs in salt marsh eggs suggests a higher dietary dependence on low-quality detritus^31^. Unexpectedly, the EPA:DHA ratio of eggs from the mangrove are similar to those from the dock habitat. This may suggest that crabs in the mangrove habitat consume more animal material than previously thought. Many studies of *A. pisonii* diet in the mangrove have focused on visual inspection of gut contents. As *A. pisonii* primarily consume only the easily digestible, difficult to identify soft parts of animals^17^, this could lead to an under estimate of the importance of animal material to their diet. Alternatively, this result may suggest that crabs on docks are feeding on a diet high in plant material as has previously been suggested for those in the mangrove. However, given previous results suggesting a diet high in animal material on docks^8^, the comparatively low trophic level of crabs in the salt marsh who have abundant access to plant material, and personal observation of crabs often feeding on dock fouling community animals such as sponges and isopods (pers. obs.), we find this explanation less likely. Ultimately, methods such as stable isotope analyses or metabarcoding of the gut contents may provide a more accurate picture of the diet of these crabs. While this topic merits further examination, it was beyond the scope of this study.

Diet appears to be the most important measured factor affecting offspring quality in this system. However, other environmental factors which differ between habitats likely also influence *A. pisonii* reproductive potential. Crabs in the salt marsh experience higher temperatures during the reproductive season than conspecifics in either the mangrove or dock habitats^8^. High development temperatures can alter the biochemical makeup^45^ and development^46^ of crustacean larvae and may increase larval metabolic rates and use of yolk reserves. This could translate to lower starvation resistance and dispersal ability upon hatching. The larger size of crabs on docks, likely a result of interacting factors^8^, further increases the quantity of the offspring they produce and, to some extent, the quality of the reproductive investment (Ω-3:Ω-6 ratio). The importance of these effects to the reproductive potential of the population is not yet known. At the extreme range edge, there may be a source-sink dynamic with docks acting as a source, especially after winter die-offs^23^. Even in areas of the salt marsh ecosystem where populations are firmly established, the higher reproductive potential of crabs on docks may allow them to have a disproportional impact on the population and may serve to accelerate repopulation after an extreme event, such as a tropical storm^25^ or an extreme winter^23^. However, further analyses would be required to determine population-level effects. Nevertheless, results here suggest that individuals on docks make an important reproductive contribution to the expanding range of this species.

While the mechanism of greatest importance may change from system-to-system, this study suggests that analogous habitats provide a suite of conditions that can increase reproductive potential. Given the importance of reproduction to colonization success^10,11^ and the relatively small area of analogous habitats within colonized ecosystems, individuals occupying analogous habitats could play vital roles in the persistence and continued expansion of shifting species. Habitat analogues may even accelerate the rate of expansion through the production of more and/or higher quality offspring, an effect that could be enhanced if the habitat also allows for increased geographic penetration into the colonized ecosystem^23^. Thus, understanding the role of analogous habitats will be critical for the management and prediction of range-shifts.

Whether they are gravel pits^47^, ponds^48^, docks^8^ or some other structure, many habitat analogues are artificial^14^ (and references therein). This provides a unique opportunity for the management of range-shifting species. The most immediate course of action is to recognize the potential of artificial structures as habitats and make a conscious effort to search them for possible range expanding species. Once such a species has been identified, direct action pertaining to the artificial habitat itself can be considered. Through the installation, alteration, or removal of analogous habitats, managers may be able to manipulate habitat effects and target reproductive hot-spots of range-shifters to encourage or reduce their spread and persistence. For instance, artificial structures could provide habitats to shifting mangrove-associated species, a habitat which is globally threatened^49^, and be used as dispersal corridors in areas of mangrove deforestation. The establishment of corridors between favorable habitats is a commonly discussed strategy to aid range-shifting species^50,51^ and artificially modified habitats have been used to improve conditions in climate-impacted native ecosystems^52,53^. However, habitat construction has not been a focus in managing the climate-mediated range-shifts of native species into new ecosystems (but see^54^). In such instances, species are not simply moving between fragments of historically-favored habitat, but colonizing entirely new ecosystems where novel habitat effects will play a permanent role in the persistence of the population. Our results suggest that the strategic placement or modification of artificial structures within these natural, but suboptimal, ecosystems could increase the reproductive success of range-shifting species that are reproduction-limited and, given the relatively small size of these habitats, play an outsized role in their persistence and rate of shift in a colonized ecosystem.

While habitat effects are likely of greatest importance to larval and seed dispersers, even mobile adult-dispersing species could receive reproductive benefits from habitat analogues through mechanisms such as predation refuge or improved diet. This potential of artificial habitat analogues to mitigate negative habitat effects and increase reproductive fitness has broad applicability across systems. Despite the relative lack of study on the role of analogous habitats during range shifts (but see^55,56^), they could provide a vital reproductive boost for shifting populations encountering suboptimal conditions. If the benefits documented here are general across systems, the role of artificial habitat analogues in altering reproductive fitness could be important to the management and success of future range-shifting species. Thus, both habitat analogues and habitat effects represent understudied phenomena in range-shift ecology that merit further investigation in the study and management of range-shifts.

## Methods

### Demographics

The body size of all ovigerous females were compared between habitats using an ANOVA followed by a Tukey’s HSD test. Further, we compared the size distributions of ovigerous females in each habitat using Komlogorov-Smirnov (K-S) tests. All statistical analyses in this study were performed in R 3.1.1.

### Energetic investment

To examine reproductive effort, we randomly collected 15 individuals by hand on each of nine randomly selected days in each habitat (Table 1) over two consecutive summers (n = 135/habitat). For all aspects of this study, sites were selected based on accessibility via kayak and chosen so that sites within habitat types were as similar as possible. Salt marsh sites were at least 0.75 km from the nearest dock ensuring that crabs in the salt marsh, which rarely stray more than 25 m from a central foraging area^19^, had no interaction with docks. Crabs were immediately placed on dry ice and kept frozen until dissection. During transport, the legs of 10 crabs collected from the mangrove became detached and mixed making it impossible to reliably obtain somatic tissue weight and resulting in a sample size of 125 crabs from the mangrove. We separated the eggs and gonads from the rest of the body, dried these to constant weight at 60–70 °C, and examined GSI as the ratio of the dry weights of the reproductive and somatic tissues of each crab. Crabs were grouped by sex and reproductive stage (male, ovigerous female, and non-ovigerous female), and we separately compared the GSI of these three groups across habitat type using independent linear models (LM) with habitat type as a fixed factor. Data were pooled across years and sites for analysis as GSI did not differ between years or site in any group (p > 0.05). While no other experiment spanned multiple years, we pooled across sites within habitats for all further analyses as site, as a fixed factor, never had a significant effect. The nature of studying a range expansion makes it impossible to avoid the fact that habitat type is confounded with latitude. We initially included latitude as a fixed factor in all statistical tests. However, latitude never explained a significant amount of the variation and was thus dropped from all analyses. Values of each result across the range of latitudes included in this study can be found in Appendix S1 (Figs. S4–S10).

### Larval quality

For both measures of larval quality (starvation resistance and larval size at hatching), we took advantage of the lunar synchronization of *A. pisonii* reproduction^16^ by collecting five ovigerous females from each of three sites in each habitat (Table 1) during the week preceding the August full moon. These 45 crabs (15/habitat) were maintained at 28–30 °C in individual aquaria (22.8·15.2·16.5 cm, l·w·h) with a petri dish of 0.2 μm filtered sea water and food from their ecosystem of origin (*Spartina alterniflora* for dock and salt marsh, *Rhizophora mangle* leaves for mangrove). Food was changed every other day and water was changed daily. Crabs were checked twice daily (8am and 11 pm) for release of larvae into the water dish, which always occurred after nightfall, and no crab was housed for more than eight days before larval release.

Upon larval release, maternal crabs were dissected. We measured the carapace width and the width of the cardiac stomach to the nearest 0.1 mm. We then calculated the GW:CW of each crab. In addition, 10 larvae were transferred to individual autoclaved 13·100 mm glass culture tubes containing ~6 ml of 0.2 μm filtered sea water. These larvae (n = 150/habitat) were checked daily for survival (starvation resistance), at which time we performed a ~2 ml water change. Once all larvae died, we examined larval starvation resistance using a cox proportional hazards model (R 3.1.1, package coxme) with habitat, maternal size, and maternal GW:CW as explanatory variables for the number of days survived. We also included maternal ID as a random factor to account for non-independence of larvae from the same mother. Lastly, 10 larvae from each brood were collected at hatching, preserved in 95% ethanol, and later dried to constant weight at 60–70 °C. We compared larval dry mass between habitats using a linear mixed model (LMM) (R 3.1.1. package lme4) with the same variables used to explore starvation resistance.

### Crab collection for clutch size and egg quality analyses

We collected 20 ovigerous females by hand from each habitat (Table 1) during the week preceding the full moon each of five consecutive months throughout the *A. pisonii* reproductive season (June–October, n = 100/habitat over season). Collected crabs were immediately placed on dry ice and stored at −80 °C until dissection, at which time the size and GW:CW were determined and the whole egg mass was carefully removed. A small number of eggs (~50) were observed via microscopy to identify development stage^31^ and returned to the egg mass. The eggs of the first 10 crabs from each monthly sampling (n months = 5) in each habitat found to be carrying stage-1 non-eyed eggs were freeze-dried, stored at −80 °C, and used for lipid and glycogen analyses (see below). The eggs of the remaining 10 crabs from each monthly sampling in each habitat were used to analyze clutch size and egg energy content (see below). Unless otherwise stated, all analyses had a sample size of 50 individuals per habitat.

### Clutch size

To determine the quantitative offspring production of *A. pisonii* in each habitat, we examined clutch size. We counted the eggs (~200) in a subset of the clutch of each crab and separately dried both this subset and the rest of the clutch to a constant weight at 60–70 °C. Total clutch size was determined by dividing the mass of the full clutch by the average mass of an individual egg in this subset. Dried clutches were stored individually for later analyses.

Clutch size scales with maternal size in *A. pisonii*^7,57^, and the average size of *A. pisonii* differs between habitats^8^. We therefore first compared clutch size between habitats independent of other factors using an ANOVA followed by a Tukey’s HSD test. We then compared clutch size between habitats while controlling for differences in maternal size by obtaining the residuals of the regression of clutch size and crab size and comparing these values between habitats using an LM with habitat, month of collection, and GW:CW as fixed explanatory variables.

### Egg energy and glycogen content

We used a Parr semi micro bomb calorimeter to determine the energy content (kJ/g) of the eggs previously used to determine clutch size and compared this value between habitats using an LM with habitat, month of collection, maternal size, and GW:CW as fixed explanatory variables. Unless otherwise stated, these fixed variables were employed in all further LMs. Egg stage was also added as an explanatory factor to account for variation attributable to developmental stage. Some clutches were pooled within habitats, months, and development stage to meet the minimum mass for calorimetric analysis, resulting in sample sizes from the salt marsh of 9 in June, 8 in September, and 5 in October as well as 9 clutches from the mangrove in October.

Following the manufacturer instructions, we used a Sigma-Aldrich Glycogen Assay Kit MAK016 to determine the glycogen concentration, as percentage of egg mass, of a subset (~10 mg) of each stage-1 clutch (see below). We compared these concentrations between habitats with an LM.

### Egg lipid and fatty acid content

We examined the egg lipids of each crab collected from each habitat (10/month, n = 50/habitat) that held stage-1 non-eyed eggs. Lipids from a subset (20–40 mg) of each clutch were extracted using a modified Folch Extraction^58,59^. Egg lipid content, as percent weight, was compared between habitats using an LM. Lipids were then flushed with nitrogen and stored at −80 °C (<2 weeks).

We analyzed the diversity and quantity of the FAs of six randomly-selected egg masses from each habitat each month (n = 30/habitat; see Appendix S1 “Supplemental Methods” for detailed methods). Briefly, we modified the methods of^60^ to methylate the FAs and analyzed the samples via gas chromatography-mass spectrometry using an Agilent Technologies 6890 N Network equipped with a 30 m Restek FAMEWAX column (0.25 mm ID, 0.25 μm df) connected to an Agilent 5975 Network Mass Selective Detector. The concentration of each FA (μg FA/μg egg) was determined via dilution curves derived from a Supelco 37 Component FAME Mix (Sigma Aldrich CRM47885). While we determined the concentration of all FAs, our analyses focused on those critical to crustacean development and larval quality. This included the total Ω-3s, the individual Ω-3s EPA, DHA, and ALA, the Ω-3:Ω-6 ratio, and the HUFA concentration; all indices which correlate positively with larval quality^30,31,41,49,50^. We also explored the fatty acid trophic markers (FATM) of EPA:DHA ratio, a measure of trophic position^31,39^ and the concentration of OFA, a measure of relative detritivory^31^. We compared the concentration of the FAs, FA groups, and FATMs between habitats using individual LMs.

## Supplementary information

Appendix S1

## Data Availability

Data available from the Dryad Digital Repository (Cannizzo *et al*. 2020), 10.5061/dryad.v15dv41sg.
