# Supplementary material for: An artificial habitat increases the reproductive fitness of a range-shifting species within a newly colonized ecosystem
Source: Sci Rep. 2020 Jan 17;10:554. doi: 10.1038/s41598-019-56228-x (PMC6969167; doi:10.1038/s41598-019-56228-x)
Supplement: Supplementary file 1 — Appendix S1 [file 41598_2019_56228_MOESM1_ESM.pdf]

Appendix S1

Supplementary Material for "An artificial habitat increases the reproductive fitness of a range-shifting species within a newly colonized ecosystem"

Zachary J. Cannizzo[1*], Susan Q. Lang[2], Bryan Benitez-Nelson[2], and Blaine D. Griffen[3]

[1]Marine Science Program and [2]Geology Program, School of the Earth, Ocean, and Environment, University of South Carolina, Columbia, SC 29208

[3]Department of Biology, Brigham Young University, Provo, UT 84602

*email: cannizzz@email.sc.edu
Current institution: National Oceanic and Atmospheric Administration Office of National Marine Sanctuaries – National Marine Protected Areas Center, Silver Spring, MD 20910

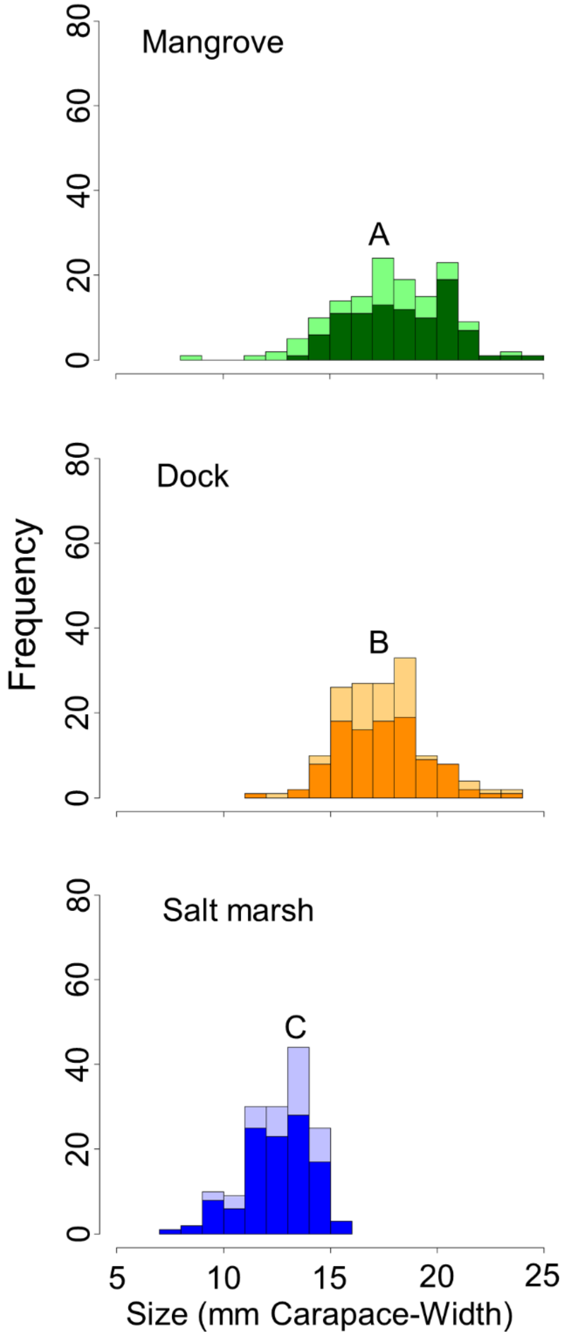

Figure S1. Size frequency distributions of ovigerous (dark colors) and all (light colors) females in each habitat. Letters represent homogeneous groups in relation to average size and size frequency distributions of ovigerous females.

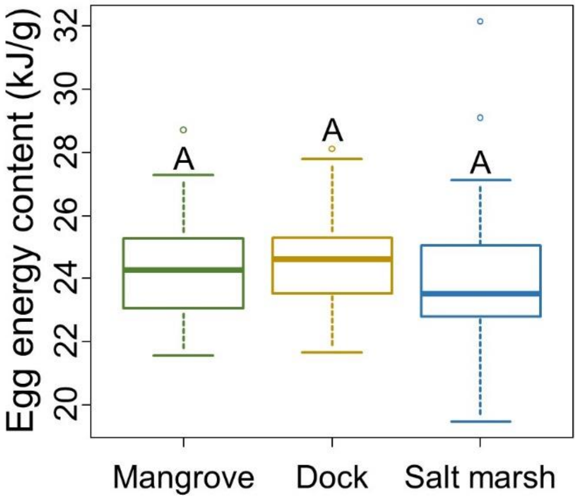

Figure S2. Gross energy content of eggs originating from each habitat.

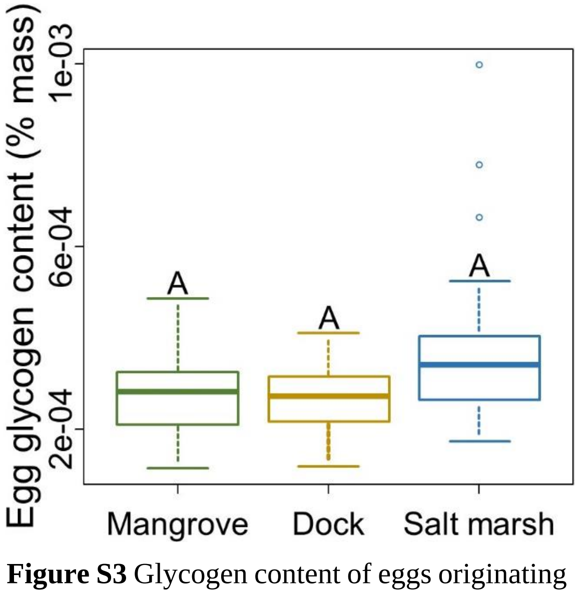

Figure S3 Glycogen content of eggs originating from each habitat.

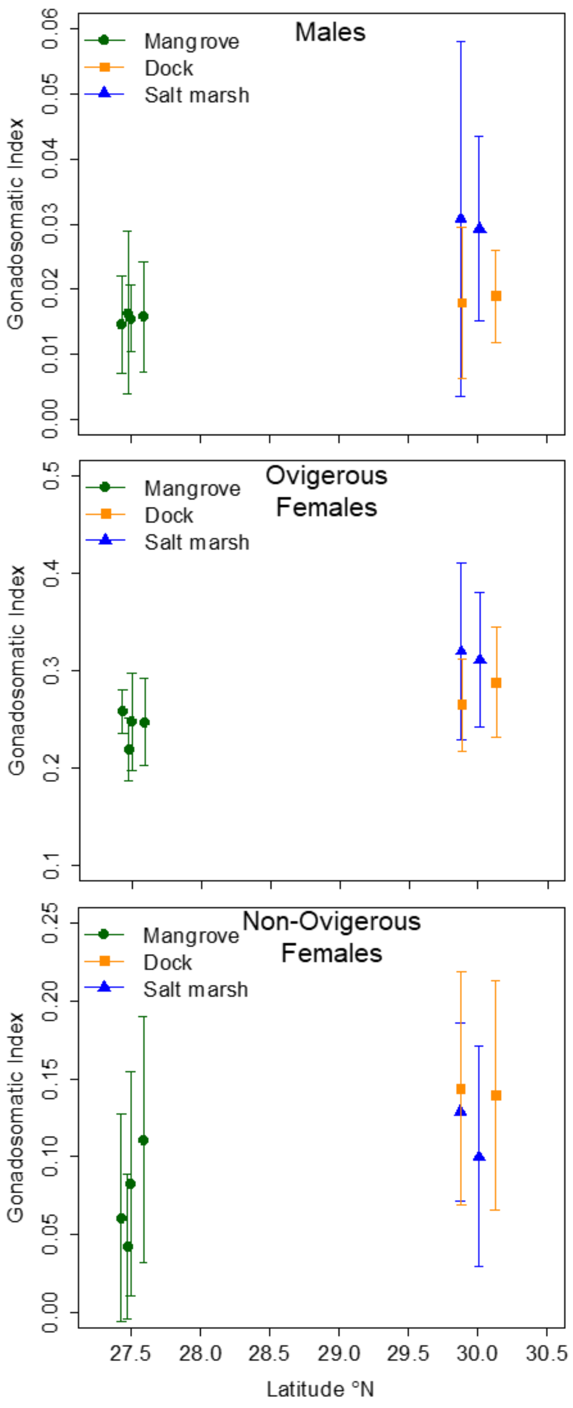

Figure S4: Average±SD proportional energetic investment into reproduction, calculated as gonadosomatic index, of male, ovigerous female, and non-ovigerous female *A. pisonii* in different habitats and across latitude

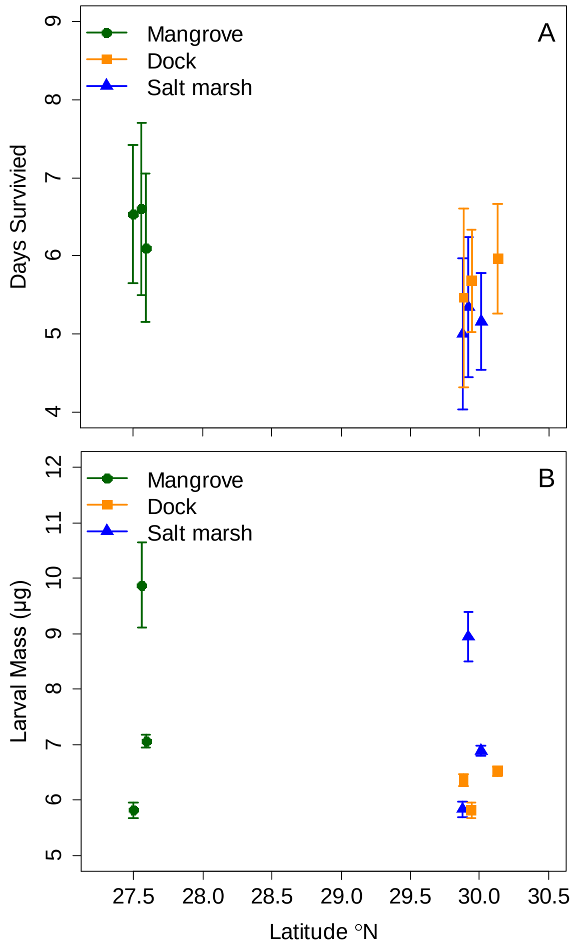

Figure S5: Average±SD of (A) larval starvation resistance measured as days survived and (B) larval size of *A. pisonii* in different habitats and across latitude

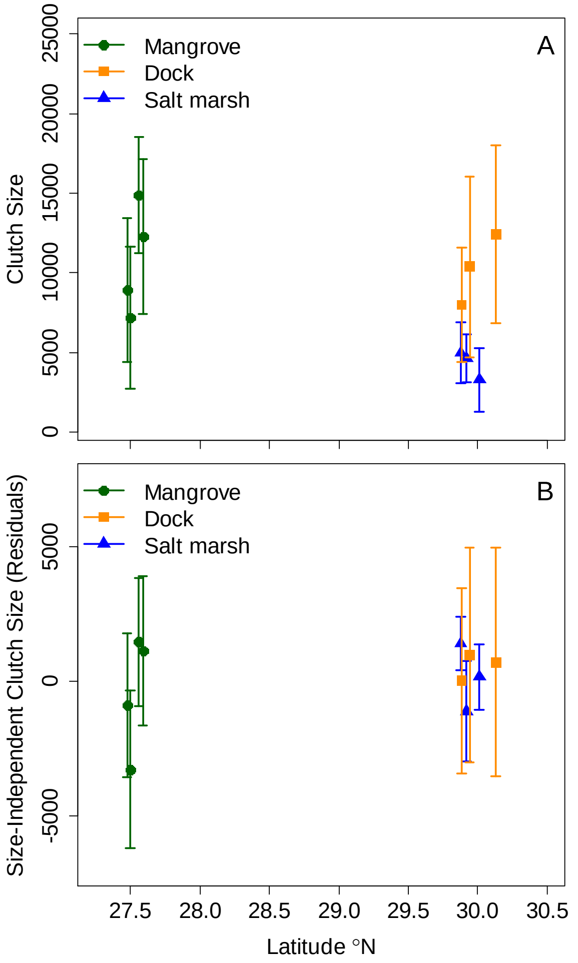

Figure S6: Average±SD of (A) *Aratus pisonii* clutch-size and (B) size-independent clutch size, represented by residuals of the relationship between clutch size and crab size (carapace-width), in different habitats and across latitude

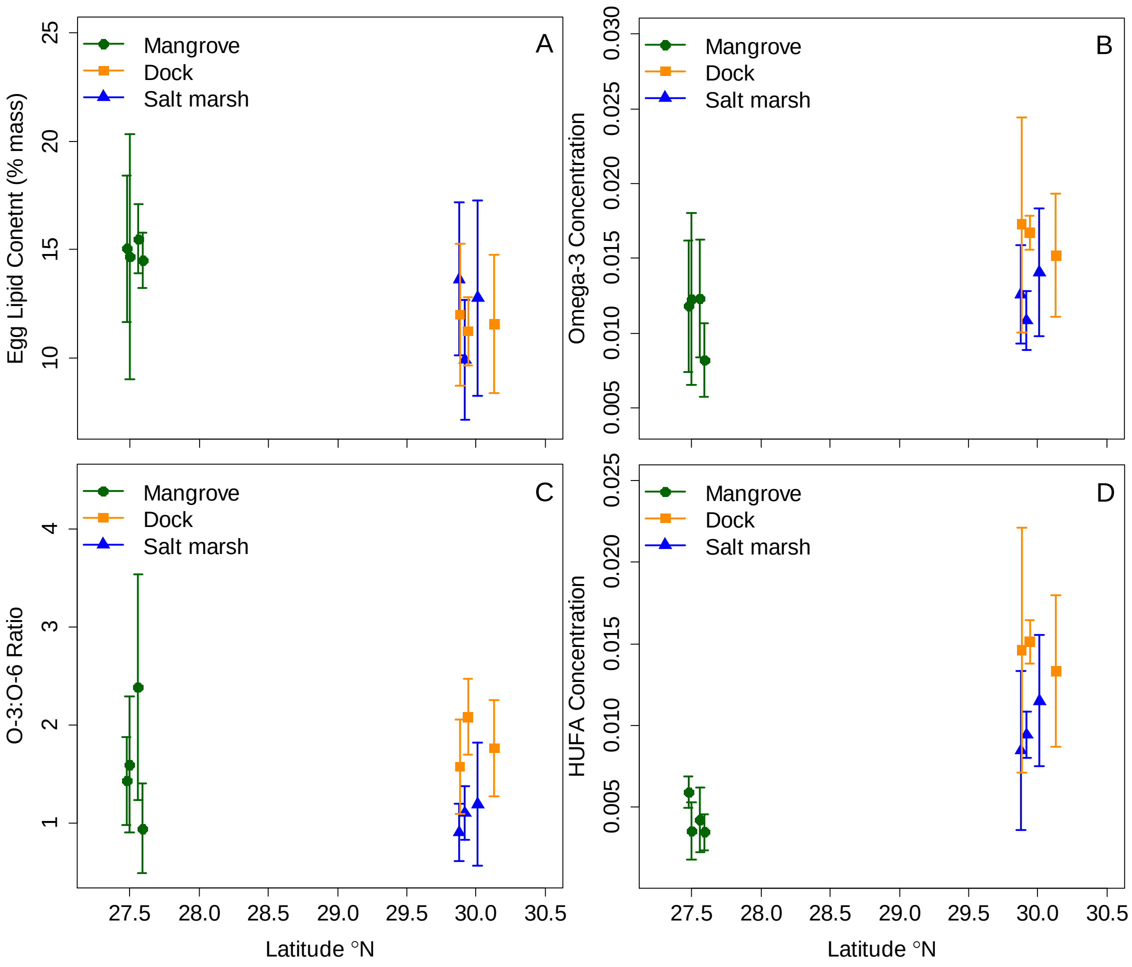

Figure S7: Average±SD of (A) gross lipid content (positively associated with larval quality) of *A. pisonii* eggs originating from each habitat and across latitude as percent of egg mass. (B) Ω-3 fatty acid content (positively associated with larval quality) of eggs originating from each habitat and across latitude as proportion of egg mass. (C) Ω-3:Ω-6 ratio (positively associated with larval quality) of eggs originating from each habitat and across latitude. Horizontal line represents a 1:1 ratio (D) Concentration of HUFA (positively association with larval quality) in eggs originating from each habitat and across latitude.

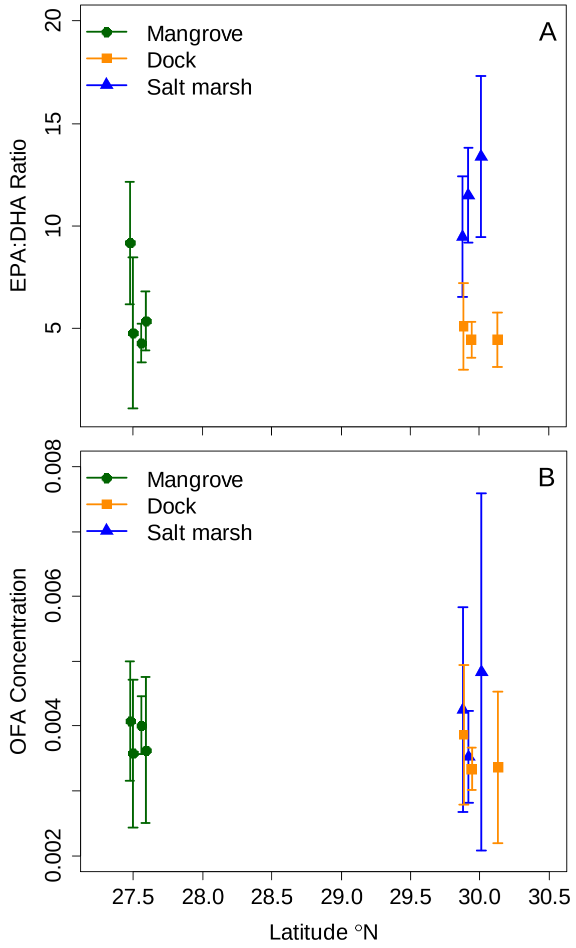

Figure S8: Average±SD of (A) EPA:DHA ratio (corresponds positively with maternal trophic level) and (B) concentration of odd-numbered fatty acids (corresponds positively with maternal relative detritivory) of eggs originating from each habitat and across latitude.

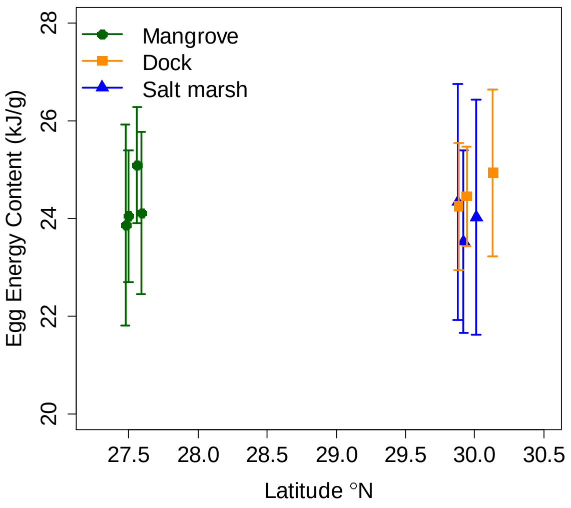

Figure S9: Average±SD of gross energy content of eggs originating from each habitat and across latitude

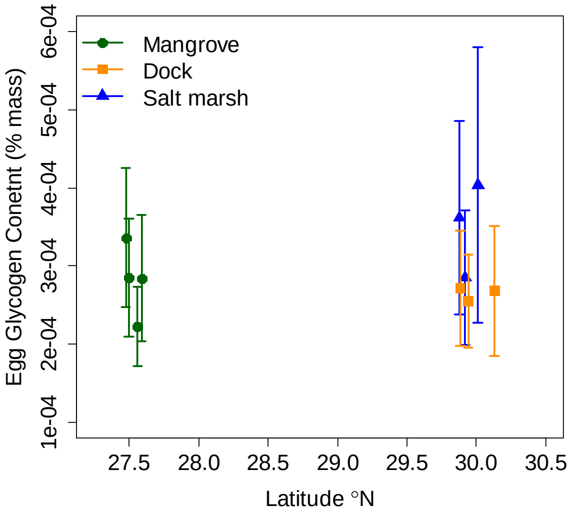

Figure S10: Average±SD of egg glycogen contant, as percent mass of eggs, across habitats and latitude

Table S1 Statistical output for results presented in the main text. Asterisks denote significant p-values (see main text)

Demographics of Ovigerous Females		
Statistical Test:	*ANOVA*	
**$F_{2,293}$**	213.6*	
Reproductive Energy Investment		
Statistical Test:	*Linear Mixed Model*	
**Explanatory Variable**	**Estimate**	**z-vale**
Males		
Mangrove vs Dock	3.097e-3	1.206
Mangrove vs Salt Marsh	0.014	5.595*
Dock vs Salt Marsh	0.011	4.281.0
Ovigerous Females		
Mangrove vs Dock	0.031	1.997*
Mangrove vs Salt Marsh	0.069	4.693*
Dock vs Salt Marsh	0.038	2.722*
Non-Ovigerous Females		
Mangrove vs Dock	0.072	4.921*
Mangrove vs Salt Marsh	0.038	2.438*
Dock vs Salt Marsh	-0.035	-2.332*
Larval Starvation Resistance		
Statistical Test:	*Cox Proportional Hazards*	
**Explanatory Variable**	**z-value**	
Maternal Size	-3.210	
GW:CW	-0.340	
Mangrove vs Dock	3.250*	
Mangrove vs Salt Marsh	5.800*	
Dock vs Salt Marsh	2.630*	
Larval Size at Hatching		
Statistical Test:	*Linear Model*	
**Explanatory Variables**	**Estimate**	**z-value**
Maternal Size	0.0119	1.241
GW:CW	25.711	2.335*
Dock vs Salt Marsh	0.201	0.280
Dock vs Mangrove	-0.219	-0.523
Mangrove vs Salt Marsh	0.0119	0.641
Clutch Size		
Statistical Test	*ANOVA*	
**$F_{2,146}$**	30.34*	
Size-Independent Clutch Size		
Statistical Test:	*Linear Model*	
**Explanatory Variable**	**Estimate**	**z-value**
GW:CW	-20689	-1.095
Mangrove vs Dock	1247.900	2.095*
Mangrove vs Salt Marsh	1239.500	2.140*
Dock vs Salt Marsh	-8.404	-0.015

July vs. June	-2395.200	-3.262
July vs August	-2305.890	-3.104*
October vs June	1539.400	2.044*
October vs July	3934.600	2.184*
October vs August	1628.700	2.184*
October vs September	2747.4	3.649*
Egg Energy Content		
Statistical Test:	*Linear Model*	
**Explanatory Variable**	**Estimate**	**z-value**
Maternal Size	0.026	0.415
GW:CW	3.645	0.349
Non-eyed vs Eyed Eggs	1.619	5.526*
Dock vs Mangrove	0.013	-0.037
Dock vs Salt Marsh	-0.321	-0.743
Mangrove vs. Salt Marsh	-0.304	-0.633
Gross Lipid Content		
Statistical Test:	*Linear Model*	
**Explanatory Variable**	**Estimate**	**z-value**
Maternal Size	-0.113	-0.755
GW:CW	-1.712	-8.151
October vs June	-4.391	-4.928*
October vs August	-3.379	-3.702*
Mangrove vs Salt Marsh	-2.565	-3.774*
Mangrove vs Dock	-3.442	-5.064*
Dock vs Salt Marsh	3.450	0.352
Omega-3 Fatty Acid Concentration of Eggs		
Statistical Test:	*Linear Model*	
**Explanatory Variable**	**Estimate**	**z-value**
Dock vs Mangrove	-0.005	-4.044*
Dock vs Salt Marsh	-0.003	-3.628*
Mangrove vs Salt Marsh	0.001	1.321
EPA Concentration of Eggs		
Statistical Test:	*Linear Model*	
**Explanatory Variable**	**Estimate**	**z-value**
Dock vs Mangrove	-7.062e-3	-8.997*
Dock vs Salt Marsh	-3.004e-3	-2.913*
Mangrove vs Salt Marsh	4.085e-3	3.458*
October vs June	-2.873e-3	1.994*
DHA Concentration of Eggs		
Statistical Test:	*Linear Model*	
**Explanatory Variable**	**Estimate**	**z-value**
Dock vs Mangrove	-1.802e-3	-8.769*
Dock vs Salt Marsh	-1.574e-3	-5.831*
Mangrove vs Salt Marsh	2.277e-4	0.741
ALA Concentration of Eggs		
Statistical Test:	*Linear Model*	

Explanatory Variable	Estimate	z-value
Mangrove vs Dock	-3.999e-3	-5.139*
Mangrove vs Salt Marsh	-2.643e-3	-2.271*
Dock vs Salt Marsh	1.355e-3	1.325
HUFA Concentration of Eggs		
Statistical Test:	*Linear Model*	
**Explanatory Variable**	**Estimate**	**z-value**
Dock vs Mangrove	-9.793e-3	-8.778*
Dock vs Salt Marsh	-4.677e-3	-3.191*
Mangrove vs Salt Marsh	5.120e-3	3.097*
October vs June	-2.873e-3	-1.994*
October vs July	-3.719e-3	-2.661*
Omega-3:Omega-6 Ratio of Eggs		
Statistical Test:	*Linear Model*	
**Explanatory Variable**	**Estimate**	**z-value**
Maternal Size	0.061	2.6088*
Mangrove vs Dock	0.303	1.803
Mangrove vs Salt Marsh	-0.146	-0.580
Dock vs Salt Marsh	-0.449	-2.032*
EPA:DHA Ratio of Eggs		
Statistical Test:	*Linear Model*	
**Explanatory Variable**	**Estimate**	**z-value**
Salt Marsh vs Dock	-6.296	-5.900*
Salt Marsh vs Mangrove	-4.847	-3.991*
Dock vs Mangrove	1.450	1.785
OFA Concentration of Eggs		
Statistical Test:	*Linear Mixed Model*	
**Explanatory Variable**	**Estimate**	**z-value**
GW:CW	-0.021	-2.340*
Salt Marsh vs Dock	-1.210e-3	-2.391*
Mangrove vs Dock	-8.230e-4	1.429
Mangrove vs Salt Marsh	-3.874e-4	1.006
Egg Glycogen Content		
Statistical Test:	*Linear Mixed Model*	
**Explanatory Variable**	**Estimate**	**z-value**
Maternal Size	-1.551e-5	-3.703*
GW:CW	-1.252e-4	-0.508
Dock vs Mangrove	3.478e-5	1.620
Dock vs Salt Marsh	2.405e-5	0.841
Mangrove vs Salt Marsh	-1.074e-5	-0.328

Table S2 Full results of fatty acid analyses. Fatty acid name and C:Dn-x code (# Carbon atoms:# double bonds n-location of first double bond; c/t signifies cis/trans). Average weight percent of each compound per egg±SD in each habitat, with letters and colors representing homogeneous groups between habitats within each row. P-value of maternal size and GW:CW, +/- represent direction of significant effect. Effect of month (LM: $p<0.05$) represented by month number and direction of effect.

Fatty Acid	C:Dn-x	Mangrove	Dock	Saltmarsh	Size	GW:CW	Month
Myristic	14:0	0.204±0.060^{A}	0.230±0.0001^{A}	0.194±0.063^{A}	0.628	0.792	8>6; 8>7; 10>6; 10>7; 10>9
Myristoleic	14:1	0.017±0.021^{A}	0.014±0.016^{A}	0.009±0.012^{A}	0.319	0.948	---
Pentadecanoic	15:0	0.100±0.033^{A}	0.081±0.036^{B}	0.119±0.059^{A}	0.816	0.011 (-)	10>6; 10>9
cis-10- Pentadecanoic	15:1	0.032±0.017^{A}	0.022±0.015^{A}	0.033±0.035^{A}	0.469	0.155	10>9
Palmitic	16:0	3.350±0.833^{A}	3.047±0.750^{A}	3.103±0.931^{A}	0.869	0.628	10>6; 10>7
Palmitoleic	16:1	1.993±0.713^{A}	1.830±0.604^{A}	1.612±0.548^{A}	0.248	0.389	10>7
Heptadecanoic	17:0	0.105±0.047^{A}	0.126±0.036^{A}	0.148±0.080^{A}	0.412	0.084	9>6
cis-10- Heptadecanoic	17:1	0.140±0.048^{A}	0.127±0.044^{A}	0.133±0.077^{A}	0.112	0.145	---
Stearic	18:0	0.466±0.141^{A}	0.468±0.140^{A}	0.535±0.429^{A}	0.222	0.839	10>6; 10>7; 10>8
Oleic/Elaidic	18:1n9c/t	0.868±0.228^{A}	0.438±0.119^{B}	0.417±0.141^{B}	0.617	0.328	10>6
Linoleic	18:2n6c	0.641±0.253^{A}	0.643±0.237^{A}	1.042±0.564^{A}	0.102	0.706	9>6; 9>8
Linolelaidic	18:2n6t	0.011±0.016^{A}	0.030±0.049^{A}	0.035±0.076^{A}	0.877	0.320	---
gamma-Linoleic	18:3n6	0.012±0.046^{A}	0.041±0.061^{A}	0.047±0.078^{A}	0.944	0.366	---
alpha-Linoleic	18:3n3	0.793±0.401^{A}	0.397±0.149^{B}	0.474±0.215^{B}	0.621	0.397	---
Arachidic	20:0	0.023±0.050^{A}	0.020±0.046^{A}	0.029±0.068^{A}	0.941	0.250	---
cis-11-Eicosenoic	20:1n9	0.021±0.009^{A}	0.025±0.017^{A}	0.018±0.018^{A}	0.403	0.389	10>6; 10>7; 10>8; 10>9
cis-11,14-Eicosadenoic	20:2n6	0.020±0.010^{A}	0.031±0.018^{B}	0.030±0.011^{B}	0.676	0.906	9>6
cis-8,11,14-Eicosatrienoic	20:3n6	0.021±0.047^{A}	0.019±0.012^{A}	0.034±0.051^{A}	0.951	0.090	---
cis-11,14,17-Eicosatrienoic	20:3n3	0.032±0.023^{A}	0.025±0.008^{A}	0.022±0.010^{A}	0.110	0.602	9>6
Arachidonic	20:4n6	0.099±0.041^{A}	0.201±0.086^{B}	0.217±0.126^{B}	0.176	0.177	9>6
cis-5,8,11,14,17-Eicosapentaenoic	20:5n3	0.258±0.128^{A}	0.981±0.384^{B}	0.703±0.301^{C}	0.642	0.747	10>6
cis-4,7,10,13,16,19-Docosahexaenoic	22:6n3	0.055±0.031^{A}	0.230±0.118^{B}	0.066±0.036^{A}	0.612	0.692	---

Supplemental Methods

Sample Storage

Unless otherwise stated, all materials used throughout these methods were acid-washed and all glass was combusted. After being removed from the pleopods of the mother (see Methods section of main paper), the whole egg clutch was placed in a glass vial with a Teflon cap. The sample was then freeze-dried and stored at -80°C until analysis.

Lipid Extraction

Lipids were extracted from the eggs using a modified Folch extraction on a 0-40mg subset of eggs [1-3]. The remaining egg clutch was returned to -80°C storage. During extraction, a known quantity of C24 alkane was added as a recover standard. Finally, at the end of the extraction the combined lipid/solvent mixture was then placed under a steady stream of Nitrogen gas to evaporate the solvent. Once dried, the remaining lipid was weighed to obtain the gross lipid mass. The vial was then capped with nitrogen and stored at -80°C until fatty acid methylation could be performed (< 2 weeks).

Fatty Acid Methylation

The fatty acids in the extracted lipids (see above) were methylated through a modification of the methods of [4]. The sample was first thawed to room temperature while a 0.5 N KOH solution was prepared by dissolving 28mg of KOH in 1ml of methanol. 1ml of 0.5N KOH was added to the sample which was then vortexed for ~30s. The sample was then placed in a sand bath and heated for 15min at 70°C. After being removed from heat, 1ml of 14% Boron Triflouride was added to the sample which was then returned to the sand bath for 5min. The sample was then allowed to cool to room temperature at which time 2ml of HPLC-grade hexane and 2ml of ultra-pure water, filtered with a Milli-Q Reference A+ system, was added. This mixture was vortexed for ~30s and allowed to stand until it separated into two layers. While the sample may be separated with a centrifuge, it was not necessary for this study. The top (lipid/solvent) layer was then transferred to a new vial and capped with a Teflon cap. The hexane-water wash was repeated twice more on the remaining water layer combining the lipid/solvent layers after each wash. The combined hexane-lipid layer was then capped with nitrogen and stored at -80°C until volume correction (<48 Hours).

For volume correction, the sample was first thawed to room temperature at which time its volume was reduced to ~2ml by evaporating the solvent under a stream of Nitrogen gas. Next, the sample was transferred at a volumetric flask and HPLC-grade hexane was added until the sample was brought to 5ml. The sample was then transferred back to its vial and stored at -80°C until interrogation via gas chromatography-mass spectrometry (GC-MS).

Sample Analysis via GC-MS

After being thawed, 2µl of the fatty acid methyl ester-hexane solution was interrogated via GC-MS on an Agilent Technologies 6890N Network equipped with a 30m Restek FAMEAX column with a 0.25mm internal diameter and a 0.25µm film thickness. This was connected to an Agilent 5975 Network Mass Selective Detector (electron ionization mode at 70eV and full scan mode 50-400 t 1.1 scans s^{-1}). The oven temperature was programed for an initial temperature of 70°C followed by a linear increase to 200°C at 10°C min^{-1} and a second linear increase at 4°C min^{-1} until 250°C which was held for 10 min. The injector was set at 250°C with the transfer line at 225°C. Helium was used as the carrier gas at a flow rate of 1ml min^{-1}.

The fatty acids (FA) were then identified by comparing retention times to known FAs of a Supelco 37 Component FAME Mix. The peak area of each FA was obtained using Agilent ChemStation software. The concentration of each FA was then calculated from a dilution curve created from the Supelco 37 Component FAME Mix spiked with a known concentration of recovery standard. The FA peak areas were first normalized to recovery standard then concentrations were determined using the regression curves of the external standards.

Works Cited

1. Folch, J., Lees, M. & Sloane-Stanley, G. H. A simple method for the isolation and purification of total lipids from animal tissues. *J. Biol. Chem.* **226,** 497-507 (1957).

2. Hara, A. & Radin, N. Lipid extraction of tissues with a low toxicity solvent. *Anal. Biochem.* **90,** 420-426 (1978).

3. Undeland, I., Härröd, M. & Lingnert, H. Comparison between methods using low-toxicity solvents for the extraction of lipids from herring (*Culpa harengus*). *Food Chem.* **61,** 355-365 (1998).

4. Morrison, W. R. & Smith, L. M. Preparation of fatty acid meth esters and dimethylacetals from lipids with boron fluoride methanol. *J. Lipid Res.* **5,** 600-608 (1964).
